# Evaluating Knowledge, Attitudes and Practices Related to Water, Sanitation and Hygiene (WASH): A Case Study of Durban High Schools in South Africa

**DOI:** 10.3390/ijerph23010061

**Published:** 2025-12-31

**Authors:** Magareth Thulisile Ngcongo, Memory Tekere

**Affiliations:** 1 Department of Environmental Sciences, University of South Africa, Science Campus, P.O. Box X6, Florida 1710, South Africa; 2 Department of Environmental Health, Mangosuthu University of Technology, Jacobs, Umlazi KwaZulu-Natal, P.O. Box 12363, Durban 4026, South Africa

**Keywords:** WASH, knowledge, attitudes and practices, school hygiene, high school learners, hygiene behaviour, South Africa

## Abstract

Inadequate hygiene knowledge and poor sanitation practices remain key challenges to safe learning environments in South Africa, with 462 million learners attending schools without basic handwashing facilities and many schools failing to meet sanitation standards. Although national policies and infrastructure investments have improved water, sanitation, and hygiene (WASH) services in some schools, access and behaviours remain uneven across socio-economic contexts. This study evaluated knowledge, attitudes, and practices (KAP) related to WASH among 1200 learners from 40 high schools in Durban using a cross-sectional design and interviewer-administered questionnaires. Data were analysed using descriptive statistics, Pearson correlations, ANOVA, and multiple regression. The study addressed the research question: To what extent do learners’ knowledge and attitudes predict hygiene practices across socio-economic contexts? It was hypothesised that higher knowledge and more positive attitudes would significantly predict improved hygiene practices. Results showed that while 74.6% reported handwashing after toilet use, only 39.3% consistently used soap. Knowledge of disease transmission through unsafe water was 35.4%, although overall attitudes were positive. Learners from higher-quintile schools had significantly better KAP scores than those from lower quintiles (*p* < 0.001). Both knowledge (β = 0.232, *p* < 0.001) and attitudes (β = 0.266, *p* < 0.001) were significant predictors of learners’ hygiene practices. Significant group differences were also observed by gender (t = 18.032, *p* = 0.001) and district (t = −3.895, *p* = 0.001). These findings highlight persistent WASH gaps and inequities across schools, underscoring the need for integrated interventions that strengthen both hygiene education and school infrastructure to achieve Sustainable Development Goal 6.

## 1. Introduction

Inequities in access to safe water, sanitation, and hygiene (WASH) remain a critical barrier to health and education in low- and middle-income countries. These disparities, often driven by socio-economic inequalities and infrastructural deficits, hinder schools, especially those in under-resourced communities, from providing adequate WASH services. In sub-Saharan Africa, many schools lack safe water, functional sanitation, and basic hygiene facilities [1,2]. In South Africa, despite policy reforms and investment in school infrastructure, WASH access continues to vary markedly across socio-economic quintiles [3]. For instance, in South Africa, despite recent policy reforms and investments aimed at improving school infrastructure, access to WASH services remains uneven across socio-economic quintiles. Schools are categorised into five quintiles, with Quintile 1 representing the most disadvantaged. This national quintile ranking system, while intended to promote funding equity, has not fully resolved service gaps in lower-quintile schools. These ongoing disparities compromise learners’ hygiene practices, elevate their risk of illness, and undermine efforts to ensure a safe and supportive learning environment [4,5].

### 1.1. Rationale for Study Setting: Durban High Schools

South Africa has implemented several policy measures to improve WASH access in schools, including infrastructure upgrades, hygiene education campaigns, and periodic monitoring. These initiatives align with Sustainable Development Goal 6 (SDG 6) [6] and the National Environmental Health Norms and Standards for Premises [7], which outline minimum requirements for school sanitation, water supply, and hygiene resources. However, despite these ongoing interventions, inequities in service quality and behavioural outcomes remain evident, particularly in lower-quintile schools [3,8].

Recent scholarship has also documented local WASH challenges specific to eThekwini Municipality. A study by [9] examining the localisation of SDG 6 targets highlighted persistent spatial inequalities, uneven service delivery, and financial constraints, despite Durban’s reputation as a progressive municipality. Another study [10] found that socio-demographic characteristics, including income, gender, and educational context, shape access to improved sanitation in eThekwini. Collectively, these findings demonstrate that WASH inequalities in Durban reflect broader national patterns of infrastructural and socioeconomic disparities.

The present study focuses specifically on high schools in Durban because the city represents a diverse socio-economic and infrastructural landscape, encompassing urban, township, rural, and informal settlement environments. These contrasting settings present meaningful variation in WASH infrastructure availability, service quality, and hygiene behaviour, making Durban an appropriate context to explore how environmental inequities shape learner WASH knowledge, attitudes, and practices. While findings offer insight into wider patterns of WASH inequality in South African schools, they remain context-specific and are not intended to serve as national generalisations.

### 1.2. Literature Review

Globally, the scale of the challenge is significant. According to the United Nations International Children’s Emergency Fund (UNICEF) and the World Health Organisation (WHO), more than 462 million children worldwide attend schools without basic handwashing facilities, while 1 in 3 schools lack safe drinking water services [11]. In sub-Saharan Africa, these deficits are even more pronounced, where large numbers of learners face daily exposure to unsafe water, inadequate sanitation, and poor hygiene [12]. This situation highlights how structural inequities in WASH provision directly affect school-age children, particularly in disadvantaged contexts [13].

In South Africa, recent years have seen policy reforms and investments aimed at improving school infrastructure, including the provision of WASH services. Schools are categorised into five quintiles, with Quintile 1 representing the most disadvantaged [14]. This national quintile ranking system was designed to promote funding equity, and it has contributed to some progress in addressing historical infrastructure gaps [15]. However, the system has not fully resolved service disparities, and lower-quintile schools continue to experience the most severe WASH-related challenges [8].

Despite recognition of the importance of WASH in schools, important gaps remain in the evidence base. Existing studies have primarily focused on primary schools, with limited attention to high school learners, particularly in urban South African contexts [13,16]. In addition, research indicates that KAP do not always align [17,18,19]. Even when learners demonstrate adequate knowledge, poor practices often persist due to the absence of enabling environments such as access to soap or functional toilets (sanitation facilities) [20,21]. Conversely, positive attitudes can be undermined by behavioural fatigue or peer influence, especially in overcrowded schools with limited infrastructure [22,23,24]. These findings suggest that knowledge and attitudes alone are insufficient predictors of hygiene behaviours, highlighting the need for a more nuanced, context-sensitive understanding of KAP dynamics [25].

### 1.3. Study Gap and Aim

Recent literature demonstrates persistent disparities in access to adequate WASH services in schools across low- and middle-income countries, where limited sanitation infrastructure, unreliable water supply, and weak hygiene promotion contribute to preventable disease transmission among learners [26,27,28]. Evidence from South Africa similarly shows uneven progress, with many schools, particularly those in lower socio-economic areas, still lacking functional handwashing facilities, menstrual hygiene support systems, and privacy-compliant sanitation structures [10,29]. Studies examining learner knowledge and hygiene behaviour have reported inconsistent handwashing practices, limited understanding of disease pathways, and school environments that do not consistently enable good hygiene behaviour [30,31]. However, there remains limited empirical evidence from South African urban settings assessing how knowledge and attitudes interact with contextual factors such as infrastructure availability. The present study contributes to this gap by examining WASH-related knowledge, attitudes, and practices among high school learners in Durban, while considering socio-economic differences between school settings.

To address these gaps, the present study aimed to evaluate hygiene-related KAP among high school learners in Durban, South Africa. Using a structured questionnaire, data were collected from a diverse sample of schools to examine how learner KAP varies across quintiles, genders, and districts. The study also analysed the statistical relationships between KAP to identify key behavioural predictors. Specifically, the study addressed the research question: To what extent do learners’ knowledge and attitudes predict hygiene practices across different school quintiles, genders, and districts? Based on prior evidence, it was hypothesised that higher knowledge and more positive attitudes would significantly predict improved hygiene practices. The findings intend to inform targeted school-based WASH interventions that support both behavioural change and infrastructure improvements, thereby contributing to Sustainable Development Goal 6 (SDG 6), which promotes equitable access to safe water and sanitation for all.

## 2. Materials and Methods

### 2.1. Study Design, Research Approach and Scope

This study employed a quantitative research approach because it enabled objective measurement of learners’ hygiene knowledge, attitudes, and practices using numerical data suitable for statistical comparison. A cross-sectional study design was selected as it allows assessment of WASH-related behaviours and contextual factors at a single point in time, making it well suited for identifying existing patterns, disparities, and associations across different school settings. This approach is commonly used in school health and public health surveys where the purpose is to describe prevalence and explore relationships rather than establish causality.

This manuscript presents findings from the quantitative WASH conditions in high schools across Durban, South Africa. The study employed a cross-sectional design, allowing for an analysis of current conditions. Structured, face-to-face interviews were conducted using an interviewer-administered questionnaire specifically designed to capture a wide range of variables related to hygiene KAP.

The scope of the study was limited to public high schools (Grades 8–12) in the Umlazi and Pinetown education districts. A total of 40 schools were included using a stratified random sampling strategy to ensure representation across school quintiles and geographical settings (urban, township, rural, and informal settlement). Private and specialised schools were not included, as the study focused on institutions governed under the national public-school WASH system. Sampling procedures and eligibility criteria are further detailed in Section 2.2.

Ethical clearance for this study was obtained from the Health Research Ethics Committee (HREC) of the College of Agriculture and Environmental Sciences, University of South Africa (UNISA) (Reference: 2021/CAES_HREC/166). Informed consent was obtained from all participants before conducting the research.

#### Definition of Key Terms

To ensure clarity and consistency, key terms used in this study are defined as follows:

Handwashing facilities: A dedicated setup that provides water and hand-cleansing agents (such as soap), enabling proper hand hygiene. In this manuscript, this term replaces variations such as hygiene stations or washing points. Information on the manufacturers of the handwashing infrastructure was not available at the time of assessment, as the facilities had been installed prior to the study and procured through decentralised processes across schools.

Handwashing soap products observed at the schools were sourced from Unilever South Africa (Pty) Ltd., Durban, South Africa, as well as from other locally available brands distributed within South Africa for which detailed manufacturer information was not consistently available. Due to decentralised procurement practices, no single manufacturer was consistently used across all schools.

Toilets (Sanitation facilities): Functional sanitation units provided within a school environment for learner use, including flush toilets, ventilated improved pit latrines (VIPs), or other acceptable structures compliant with the South African National Environmental Health Norms and Standards for Premises.

WASH (Water, Sanitation, and Hygiene): A collective term referring to the infrastructure, services, and behaviours related to access to safe drinking water, adequate sanitation, and hygienic practices in school environments.

These standardised definitions were applied across the manuscript to ensure consistency in reporting.

### 2.2. Study Area and Participants

The study was conducted in the Umlazi and Pinetown education districts of eThekwini Municipality, Durban, KwaZulu-Natal Province, South Africa, selected to capture socio-economic and geographical diversity. Public high schools in these districts were stratified according to the national quintile ranking system, ranging from Quintile 1 (poorest) to Quintile 5 (wealthiest). A total of 40 schools were selected using stratified random sampling to ensure proportional representation across quintiles. A two-stage stratified random sampling method was employed, selecting schools based on both quintiles (1–5) and geographical contexts (urban, township, suburban, rural). Within each stratum, proportional sample sizes were determined:

$$n_{i}=\left(\frac{N_{i}}{N}\right)\times n$$

where $n_{i}$ is the sample size for stratum i; $N_{i}$ is the population size for stratum i; *N* is the total population size and n is the total sample size. A minimum of 28 schools was required, but this was increased to 40 for reliability.

The sampling frame comprised public ordinary high schools (Grades 8–12) listed in the Department of Basic Education’s Education Management Information System (EMIS) database. Independent, private, and specialised schools were excluded to maintain a uniform governance and funding context. Eligible institutions were therefore co-educational public schools offering Grades 8–12. Within each selected school, learners were chosen through systematic random sampling using updated class registers as the sampling list.

Learner sample size was calculated using Cochran’s formula:

$$n_{0}=\frac{Z^{2}\times \;p\times q}{e^{2}}$$

where *Z* is the Z-score corresponding to the desired confidence level (for 95%, *Z* = 1.96); *p* is the estimated proportion of success (assumed to be 0.5 for maximum variability) *q* = 1 − *p* and *e* is the margin of error (set at 0.05 for 5%).

Given the population size of 175,015 students, the sample size was adjusted using the finite population correction (FPC):

$$n=\frac{n_{0}}{1+\frac{n_{0}-1}{N}}$$

where $n_{0}$ is the initial sample size (from Cochran’s formula); *N* is the total population size and n is the adjusted sample size. the estimated sample size was 384 students. For feasibility, 30 learners were sampled per school (Grades 8–12), resulting in a final sample of 1200 learners across 40 schools.

### 2.3. Data Collection Instrument and Procedure

To assess learners’ KAP related to WASH, a structured interviewer-administered questionnaire was used. The instrument was adapted from a United Nations International Children’s Emergency Fund (UNICEF) school WASH tool [32] and contextualised to the local setting. Adaptation involved simplifying wording for age appropriateness, aligning terminology with South African WASH policies (including the National Environmental Health Norms and Standards for Premises, 2015), and adding items relevant to the school context, such as menstrual hygiene facilities and access to soap. A pilot study was conducted in two public high schools that were not part of the main sample to assess clarity, comprehension, and completion time. Feedback from this process informed minor wording adjustments and removal of redundant items. The reliability of the final questionnaire was assessed and confirmed, as described in Section 2.5 (Data Analysis). In addition to KAP, the questionnaire included items on the self-reported prevalence of symptoms potentially associated with WASH-related diseases. The final instrument consisted of sections on socio-demographic details, knowledge of WASH, attitudes towards WASH, and hygiene-related practices and symptom experience. Data was collected over a 12-month period, with seasonal observations at different sites. through individual, face-to-face interviews conducted in a secluded area within each school to ensure confidentiality and minimise distractions. The interviews were conducted by the lead researcher and two trained assistants, who read each question aloud and directly recorded the learner’s responses onto the questionnaire. No audio or electronic recording devices were used. To capture potential seasonal variation, data collection occurred quarterly, with a different group of learners sampled during each round. A total of 30 learners per school were interviewed. Direct recording of responses by the interviewer helped ensure data completeness and consistency across participants.

### 2.4. Data Quality Assurance

To ensure the accuracy and completeness of the quantitative data collected during student interviews, several quality assurance measures were implemented. All interviews were conducted face-to-face by the lead researcher and two trained research assistants using the structured interviewer-administered questionnaire. During each interview, the questionnaire was completed by the interviewer on behalf of the learner to minimise missing or inconsistent responses, with clarifications provided where needed. Upon completion of data collection, all hard-copy questionnaires were securely stored.

Data entry was performed by a trained Information Technology specialist using Microsoft Excel, followed by a double-entry verification process. The lead researcher independently re-entered 10% of the questionnaires and compared entries with the original dataset. Random spot checks and consistency reviews were also conducted to identify missing values, duplicates, and transcription errors. Any discrepancies identified during this process were corrected before the final dataset was imported into IBM SPSS Statistics version 27 for statistical analysis.

### 2.5. Data Analysis

Quantitative data were analysed using IBM SPSS Statistics version 27. Descriptive statistics, including frequencies, percentages, means, and standard deviations, were used to summarise socio-demographic characteristics and KAP responses. Internal consistency of the KAP scales was assessed using Cronbach’s alpha. Inferential statistics were used to explore differences and relationships within the data. Independent samples *t*-tests and one-way ANOVA were applied to examine differences in mean KAP scores across demographic groups such as gender, school quintile, and district. Pearson’s correlation coefficient was used to assess relationships between KAP. Multiple linear regression analysis was conducted to identify significant predictors of hygiene-related practices, using knowledge and attitude scores as independent variables. Assumptions of normality, linearity, homoscedasticity, and multicollinearity were checked prior to conducting regression analysis. These statistical tests were selected because the KAP scores were continuous and approximately normally distributed, allowing the use of parametric analyses suited to comparing group means, assessing correlations, and identifying predictors in line with the study’s research question on how learners’ knowledge and attitudes predict hygiene practices. Statistical significance was set at *p* < 0.05.

Data were checked for completeness and consistency before analysis. All questionnaires were fully completed, and no missing data were observed; therefore, all analyses were conducted on complete cases.

## 3. Results

### 3.1. Response Rate and Participant Characteristics

A total of 1200 high school learners from 40 public schools in Umlazi and Pinetown districts participated in the study, achieving a 100% response rate. Schools were proportionally selected across the five national quintiles to reflect socio-economic diversity. The socio-demographic characteristics of the participants are summarised in Table 1. Among the respondents, 53.2% were female and 46.8% male, with learners distributed across Grades 8 to 12. Most participants were between the ages of 15 and 18 years.

### 3.2. Knowledge of WASH

Learners demonstrated mixed levels of knowledge across the assessed WASH topics. Learners’ knowledge of key WASH indicators is summarised in Table 2. While 53,3% correctly identified critical handwashing times, only 25.8% recognised the importance of using soap and water, and just 41.9% knew the appropriate boiling duration to make water safe for drinking. Knowledge of proper faeces disposal was similarly low, with 46.7% answering correctly. In contrast, a stronger understanding was observed for materials used for personal hygiene, with more than half selecting appropriate responses. Gender-disaggregated analysis showed that female learners attained a higher mean knowledge score than males (*p* = 0.001), underscoring the value of gender-responsive hygiene education to close residual gaps. These results point to significant gaps in foundational WASH knowledge, particularly environmental sanitation, which may compromise safe hygiene practices among students.

### 3.3. Attitudes Toward WASH

The results on learner attitudes indicate that most learners held a generally positive attitude toward WASH, although several critical concerns were identified. A substantial majority (84.3%) recognised the importance of access to clean water, sanitation, and handwashing facilities in schools, and 90.8% agreed that school water should be safe to drink. However, only 55.9% believed that their school provided enough drinking water, and more than three-quarters (76.3%) reported that toilets were not always functional.

Concerns about privacy were also evident, with 70.3% of learners stating that the school toilets lacked sufficient privacy. While 83.0% agreed that handwashing facilities should be available in schools, only 24.5% confirmed the availability of clean water and soap. These results point to a notable disconnect between learners’ recognition of WASH importance and their perceptions of adequacy within the school environment. Table 3 presents a detailed summary of learners’ responses regarding WASH attitudes.

### 3.4. Hygiene Practices Among Learners

Most learners (74.9%) reported washing their hands after using the toilet, reflecting general awareness of personal hygiene. However, only 65.8% indicated that they cleaned up after themselves after using school toilets, pointing to inconsistencies in toilet hygiene practices. Furthermore, just 50% of participants reported consuming sufficient water while at school, suggesting a potential concern regarding hydration and access.

As shown in Table 4, participation in hygiene education was notably low, with only 34.4% of learners reporting involvement in such activities. On a positive note, most learners, 79.0% stated that they did not urinate behind school toilets, indicating a level of adherence to basic sanitation norms. Despite this, 57.8% reported that they seldom used school toilets, which may reflect discomfort, lack of privacy, or dissatisfaction with sanitation conditions. These findings reveal a gap between hygiene awareness and consistent hygiene behaviour, suggesting the need for improved hygiene education and a more supportive WASH infrastructure in schools.

Menstruation-related indicators point to constraints that shape hygiene behaviour in schools. Only 23.4% of female learners reported feeling comfortable using school toilets during menstruation, and 48.8% reported menstruation-related absenteeism. In addition, just 44.0% reported maintaining personal hygiene during menstruation while at school. Taken together with toilet avoidance among 57.8% of learners and low participation in hygiene education at 34.4%, these results indicate socio-cultural and dignity-related barriers, centred on privacy, comfort, and support, that may limit consistent hygiene practice despite general awareness.

### 3.5. Correlation Between Knowledge, Attitudes, and Practices

Standardised beta coefficients showed that both predictors were statistically significant. Knowledge had a positive effect on hygiene practices (β = 0.23, *p* < 0.001), indicating that an increase in knowledge was associated with improvement in hygiene-related behaviour. Attitudes also demonstrated a significant positive effect (β = 0.27, *p* < 0.001). This means that for every one-unit increase in the knowledge score, the hygiene practice score increased by 0.23 units, whereas a one-unit increase in the attitude score resulted in a 0.27-unit increase in hygiene practice.

No significant interaction was detected between knowledge and attitudes, indicating that their influence on hygiene practices operates independently rather than synergistically. This aligns with the weak but statistically significant correlation between knowledge and attitudes shown in Table 5 (r = 0.15, *p* < 0.001). The correlation matrix demonstrates that both knowledge (r = 0.27, *p* < 0.001) and attitudes (r = 0.30, *p* < 0.001) are individually associated with hygiene practices.

Collectively, these findings highlight that knowledge and attitudes function as separate yet complementary determinants of hygiene behaviour, reinforcing the need for WASH interventions that address both cognitive understanding and motivational drivers.

### 3.6. Association of Demographic Characteristics with KAP

Association between learners’ demographic characteristics and their mean scores for KAP related to WASH is summarised in Table 6. The means (M) and standard deviations (SD) are reported alongside statistical tests (*t*-tests and ANOVA) to assess differences across gender, age, grade, quintile, and district. A statistically significant gender difference was observed in both knowledge and practice scores. Female learners demonstrated higher knowledge (M = 0.50, SD = 0.21) and better hygiene practices (M = 0.60, SD = 0.19) than male (knowledge: M = 0.44, SD = 0.21; practice: M = 0.40, SD = 0.16), learners, with both differences being significant (*p* = 0.001). However, no significant difference was found in attitudes between genders (*p* = 0.42).

Attitudinal differences were also observed across age groups. Although knowledge and practice scores did not differ significantly by age, a descriptive trend was observed in hygiene practices across age groups. Learners aged 12–15 years demonstrated the highest hygiene practice scores (M = 0.53, SD = 0.21), followed by those aged 16–19 years (M = 0.51, SD = 0.20), while learners aged 20–24 years reported slightly lower hygiene practice scores (M = 0.500, SD = 0.22). Despite this pattern, these differences were not statistically significant (*p* = 0.14). Learners aged 12–15 years reported more positive attitudes (M = 0.61) compared to their older counterparts. This suggests that younger students may be more receptive to hygiene messaging, though this did not translate into significantly different knowledge or practice scores. In terms of school grade, statistically significant differences were found in knowledge and attitudes, *p* = 0.004 and *p* = 0.001, respectively. Grade 11 learners had the highest knowledge (M = 0.51) and most positive attitudes (M = 0.62). Although the variation in practice across grades was only marginally significant (*p* = 0.06), Grade 9 learners reported the highest level of hygiene practice (M = 0.54), indicating a possible trend.

Significant differences in KAP scores were observed across school quintiles (knowledge: F = 25.02, *p* = 0.001; attitudes: F = 15.04, *p* = 0.001; practices: F = 18.18, *p* = 0.001). Learners in Quintile 5 schools had the highest knowledge (M = 0.54), attitudes (M = 0.63), and practices (M = 0.58), highlighting the influence of socioeconomic status on WASH-related behaviour. Lastly, district-level differences were evident in knowledge and practice. Umlazi learners reported significantly higher knowledge (M = 0.50) and practice (M = 0.54) scores than those in Pinetown (*p* = 0.001). However, no significant difference in attitudes was observed between the districts (*p* = 0.40). These findings highlight key demographic disparities that could inform targeted WASH interventions. These patterns align with SDG 6 equity indicators for school WASH, signalling gaps in equitable access and service conditions that require targeted improvement.

Post hoc pairwise comparisons using the Bonferroni correction indicated that Grade 9 learners had significantly higher hygiene practice scores compared with Grade 8 learners (*p* < 0.05). Significant differences were also observed between Grade 11 and Grade 12 learners (*p* < 0.05), where Grade 11 reported higher practice scores. No other pairwise comparisons between grade levels were statistically significant (Table 7).

### 3.7. Multiple Regression Analysis

**Figure 1:**
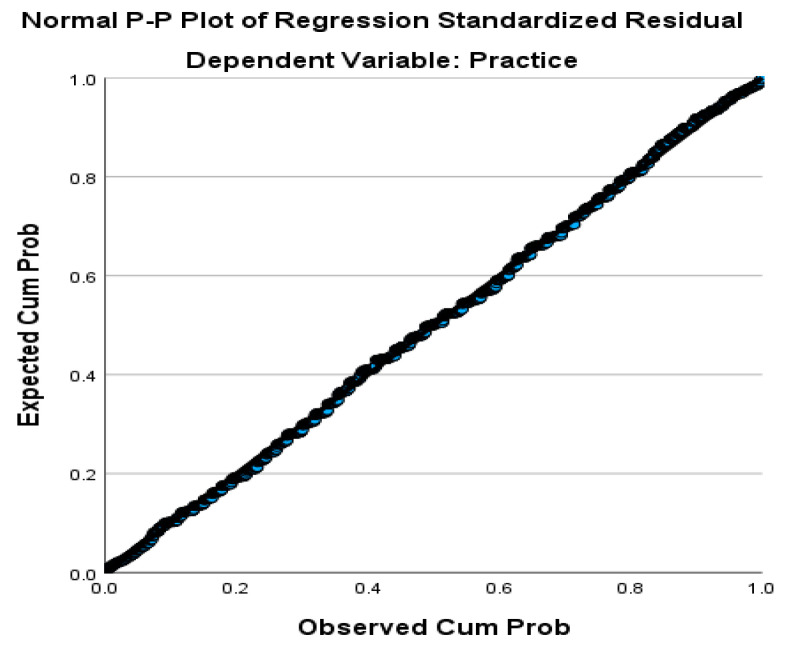
Normal P–P plot of standardised regression residuals.

Multiple regression analysis was used to examine whether knowledge and attitudes significantly predict good hygiene practices among learners. Prior to analysis, assumptions of normality, linearity, homoscedasticity, and multicollinearity were evaluated. Normality of the data was supported by skewness values falling within the acceptable range of +1 to −1 [33], and a normal probability plot (P–P) of regression standardised residuals showed that points aligned closely along the diagonal line, indicating no major deviation from normality (Figure 1). Linearity and homoscedasticity were confirmed through inspection of the residual scatter plot, which showed a roughly rectangular pattern and evenly distributed residuals [34]. These results indicate that the assumptions for multiple regression were met.

This plot compares the observed cumulative probability of residuals with the expected cumulative probability under a normal distribution. The close alignment of points along the diagonal line indicates that the assumption of normally distributed residuals was met.

To assess multicollinearity, bivariate correlations were examined alongside tolerance and variance inflation factor (VIF) statistics. Correlation coefficients between the independent variables were below the 0.90 threshold, and tolerance values exceeded 0.10, while VIF values were below 10.0 (Table 8), confirming that multicollinearity was not a concern [35]. Therefore, both predictors, knowledge and attitudes, were retained in the final regression model.

The regression model was statistically significant F (2. 12) = 100.25, *p* < 0.001) and explained 14.3% of the variance in good hygiene practices (R^2^ = 0.14). The overall correlation between the predictors and the dependent variable was moderate (r = 0.38), suggesting a meaningful but not exhaustive relationship (Table 8).

Standardised beta coefficients showed that both predictors were significant. Knowledge had a positive effect on hygiene practices (β = 0.23, *p* < 0.001), indicating that an increase in knowledge was associated with an improvement in hygiene-related behaviours. Attitudes also showed a significant positive effect (β = 0.27, *p* < 0.001), and this relationship was slightly stronger than that of knowledge. These findings underscore the importance of both cognitive and affective components in shaping WASH behaviours.

In summary, the regression results confirm that both knowledge and attitudes are significant predictors of hygiene practices. While the explained variance is moderate, these findings highlight the potential of education and awareness programmes in enhancing hygiene behaviours, while also pointing to the need for future research exploring additional influencing factors.

**Figure 2:**
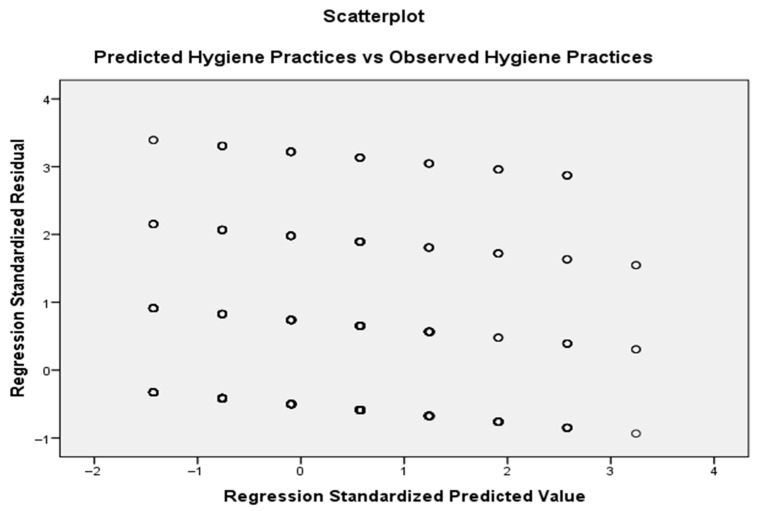
Scatterplot of standardised residuals versus standardised predicted hygiene practice scores.

Model diagnostics including the standardised residuals scatterplot (Figure 2), were examined to assess model fit. The residuals appeared randomly dispersed without a clear pattern or funnel shape, indicating that the assumptions of linearity and homoscedasticity were met, supporting the adequacy of the regression model.

This scatterplot assesses the linearity and homoscedasticity assumptions of the regression model. The random dispersion of points around zero, with no visible clustering pattern or funnel shape, suggests constant variance and supports the suitability of the regression model.

For every one-unit increase in knowledge score, hygiene practice scores increased by 0.23 units (*p* < 0.001). Similarly, a one-unit increase in attitude score was associated with a 0.27-unit increase in hygiene practice score (*p* < 0.001). No significant interaction was detected between knowledge and attitudes, indicating that their effects on hygiene practice operate independently.

### 3.8. Association Between Quintile and WASH Infrastructure Availability

To determine whether differences in hygiene practices across quintiles were associated with WASH infrastructure availability, a chi-square analysis was conducted. A statistically significant association was found between quintile classification and perceived adequacy of clean water and soap for handwashing (χ^2^ = 12.08, df = 4, *p* = 0.02). Learners from higher-quintile schools were more likely to report adequate handwashing infrastructure compared with those from lower-quintile schools (Table 9). These findings suggest that hygiene practice disparities are partly influenced by unequal access to enabling WASH infrastructure.

## 4. Discussion

This study revealed a persistent gap between learners’ knowledge of hygiene practices and their actual behaviours, reinforcing findings documented in numerous low- and middle-income countries. Although many learners demonstrated a sound understanding of sanitation and hand hygiene, this awareness did not consistently translate into practice. As reflected in the study results, about three-quarters of learners recognised the importance of handwashing with soap, yet only about half reported washing hands frequently at school, and just under two-thirds indicated cleaning up after toilet use. The lack of soap, functional handwashing facilities, and adequate toilets were frequently cited barriers to proper hygiene, mirroring findings from South Africa, Ethiopia, and Bangladesh [36,37,38].

In line with the Health Belief Model (HBM), the study underscores that behaviour change is more likely when individuals perceive a health risk as serious and have access to the necessary support and infrastructure to respond effectively [39,40]. While students recognised the health implications of poor hygiene and expressed positive attitudes toward handwashing and sanitation, systemic and infrastructural constraints limited their ability to adopt and sustain good practices. As indicated in our findings, fewer than one-third of learners perceived sufficient toilet privacy, and only about one-quarter regarded the school toilets as being in good working order, illustrating how structural deficiencies weaken otherwise positive attitudes. Similarly to findings in Addis Ababa and Mogadishu, schools with handwashing facilities often lacked soap and water simultaneously, weakening the utility of existing infrastructure [41,42]. This directly impedes progress towards SDG 6, particularly target 6.2, which aims to achieve access to adequate and equitable sanitation and hygiene for all and highlights the inequities in school settings [20,21].

The findings indicate that socio-cultural barriers, such as stigma, menstruation-related norms, and privacy expectations, and dignity-related conditions within school environments substantively shape learners’ hygiene behaviour. Practice signals such as avoidance of school toilets, discomfort using facilities during menstruation, and limited participation in hygiene education point to stigma, privacy concerns, and inadequate support for menstrual health management; these are consistent with contexts where prevailing norms and weak hygiene-promotion efforts fail to reinforce handwashing even among learners who recognise its importance [43]. This interpretation is further supported by the study results, which showed that fewer than one-quarter of girls felt comfortable using school toilets during menstruation, while roughly half reported missing school during that period. Together these findings highlight how gender-specific barriers and inadequate infrastructure continue to undermine menstrual-hygiene management within the school environment. Furthermore, the lack of health education programmes, hygiene coordinators, and institutional support in schools reduced the motivation and consistency required for sustained behaviour change [30,44]. Addressing these barriers requires the provision of adequate WASH facilities, gender-responsive hygiene promotion, and organisational arrangements that sustain practice through monitoring, maintenance, and staff accountability [30,44].

Socioeconomic disparities also played a critical role, as students attending higher-quintile schools generally demonstrated better hygiene practices and access to resources compared to those in lower-quintile schools. This pattern appears to be influenced by differences in WASH infrastructure availability, as the chi-square results showed that access to clean water and soap was significantly more common in higher-quintile schools. This aligns with global literature linking resource availability to hygiene outcomes [45]. Gender differences were also noted, with girls showing stronger knowledge and better practices than boys, aligning with studies that report greater hygiene consciousness among adolescent girls [31,46]. Beyond gender and school quintile, the age and grade patterns point to practical times to strengthen WASH behaviours, for example, early adolescence (12–15 years), Grade 9 when practice was highest, and Grade 11 when knowledge and attitudes were strongest. Differences between districts reflect unequal resources and the consistency of facility maintenance. Taken together, these findings highlight structural factors, school governance, day-to-day operations and maintenance, and local management capacity that shape learners’ chances to practice good hygiene. Viewed through an equity lens, these patterns align with SDG 6 monitoring [20,21] and help identify who and where to prioritise, especially lower-quintile schools and underperforming districts.

Although the regression showed that knowledge and attitudes significantly predicted hygiene behaviours, the model accounted for only a modest share of the variance (R^2^ = 0.143). This indicates that additional school- and social-level determinants play a meaningful role. Likely influences include peer norms, teacher and parental modelling, supply chain reliability for consumables, active hygiene coordinators, and school governance. Future research should examine these determinants directly and evaluate implementation strategies that strengthen them within routine school practice [47].

It is also worth noting that not all studies arrive at the same conclusions. Some research has shown that educational level can strongly influence hygiene knowledge and practices, with learners at higher grades demonstrating better behaviours. Post hoc pairwise comparisons showed that Grade 11 learners performed significantly better than Grade 8 and Grade 9 learners (*p* < 0.05), while no significant differences were observed between Grades 10, 11, and 12. This suggests that improvements in hygiene behaviour may reflect increased maturity or exposure to hygiene messaging rather than a linear progression across all school years [48]. In contrast, this study found that while knowledge and attitudes matter, they are not enough when broader systemic barriers remain unresolved. Similarly, research conducted in Bengaluru, Karnataka, India, has suggested that health education and communication campaigns can bring about lasting improvements in safe hygiene behaviours [49]. Yet in the Durban schools studied here, education on its own did not close the gap between knowledge and practice. These contrasts suggest that while education and campaigns are powerful tools, they must be reinforced by reliable infrastructure, gender-sensitive facilities, and supportive school systems if they are to deliver lasting change.

Taken together, these findings reinforce that hygiene practices among learners are shaped not only by individual knowledge and attitudes but also by broader contextual and institutional factors. The modest predictive power of knowledge and attitudes, combined with the significant disparities observed across quintiles and districts, suggests that behaviour change cannot occur in isolation from enabling school environments. This aligns with behavioural theory, which emphasises that motivation must be supported by opportunity and capability for sustained habit formation. Therefore, improving WASH outcomes requires an integrated approach that strengthens behaviour-change efforts while simultaneously addressing infrastructure gaps, resource availability, and school-level governance mechanisms that either support or constrain healthy hygiene practices.

The findings of this study also have important theoretical and practical implications. Theoretically, the results reinforce behavioural models such as the Health Belief Model and Behaviour Change Wheel, which suggest that knowledge alone is insufficient to sustain positive hygiene behaviour unless supported by motivation, environmental cues, and enabling conditions. The modest variance explained by the regression model demonstrates that hygiene behaviour is influenced by multiple interacting determinants beyond cognitive understanding. Practically, the findings highlight the need for integrated WASH interventions that go beyond awareness campaigns and ensure access to soap, functional handwashing facilities, menstrual hygiene support, and safe sanitation infrastructure. The strong association between school quintile and outcomes further indicates that interventions should prioritise lower-resourced schools and include monitoring systems that ensure consistent maintenance and a reliable supply of hygiene materials.

Although the study makes a meaningful contribution to understanding WASH practices in schools, some limitations are worth noting. The cross-sectional design does not allow causal inferences between knowledge, attitudes, and practices; however, it provides valuable insights into associations that can guide future longitudinal research. The focus on Durban high schools may also restrict the generalisability of the findings to other regions of South Africa with different resources and socio-cultural conditions. These schools are located in a metropolitan municipality with comparatively higher service coverage and administrative capacity than many rural or peri-urban areas. As such, the findings should be interpreted within the local context of eThekwini Municipality, where quintile-based inequalities, infrastructure quality, and school-governance dynamics uniquely shape WASH provision. The patterns observed may therefore differ in provinces or districts with distinct climatic, economic, or institutional environments.

In addition, the study did not include longitudinal or qualitative components that could capture behavioural changes or provide deeper contextual understanding of learners’ experiences. The absence of these methods limits the ability to explore causal relationships or understand the subjective drivers behind hygiene behaviours. Future mixed-methods or longitudinal designs could address these aspects by examining behavioural trends over time and integrating stakeholder perspectives to enrich interpretation.

The regression model accounted for a modest proportion of the variance in hygiene practices (R^2^ = 0.143), indicating that knowledge and attitudes, although statistically significant, explain only part of learners’ hygiene behaviour. This limited explanatory power suggests that other contextual factors, such as infrastructure adequacy, peer norms, household practices, and institutional support, also influence hygiene outcomes. Practically, this implies that interventions should combine educational and structural strategies to enhance behavioural outcomes. Future research could expand the model to include additional school-, home-, and community-level determinants or employ multilevel analyses to better capture the complex drivers of hygiene behaviour. Recognising these limitations underscores the need for future mixed-method and longitudinal studies to build on these findings and strengthen the evidence base.

Because the study relied primarily on self-reported data, there is a possibility of recall bias and social-desirability effects, whereby learners may have overstated desirable hygiene behaviours. The use of interviewer-administered questionnaires helped clarify questions and reduce misunderstanding, yet self-reporting remains an inherent limitation. Future research could strengthen data validity through triangulation methods such as direct observations of hygiene practices, school facility assessments, or teacher-reported behaviour tracking.

Ultimately, bridging the knowledge-behaviour gap in school hygiene requires more than awareness campaigns. This conclusion aligns with the statistical results, where knowledge and attitudes explained only about 14% of the variance in hygiene behaviour, underscoring that awareness alone is insufficient to sustain practice. There is a need for coordinated multi-pronged interventions that combine WASH education with functional infrastructure, behavioural reinforcement, and policy support. These findings align with calls for school-based hygiene strategies that are supported by institutional policies, dedicated funding, and monitoring frameworks [50,51,52]. Priority should first be given to ensuring a reliable water supply, functional toilets, and accessible hand-washing facilities with soap, as these form the foundation for sustained hygiene behaviour. Next, hygiene-promotion initiatives should be continuous and participatory, using peer-led clubs, classroom demonstrations, and teacher modelling to normalise good practices. Finally, routine monitoring and maintenance systems should be established with clear accountability assigned to school management teams in collaboration with Environmental Health Practitioners. Such targeted actions can translate knowledge into daily practice and strengthen sustainability. When embedded within a supportive environment, such efforts have the potential to improve learners’ health, dignity, and academic experience, particularly in disadvantaged school contexts. The findings should, however, be interpreted within the specific context of Durban high schools and not assumed to represent all South African school settings.

## 5. Conclusions

This study demonstrates a persistent knowledge–behaviour gap in hygiene among learners in selected high schools in Durban, with uneven knowledge across core domains and positive attitudes co-existing with perceptions of inadequate facilities. Practice patterns, including toilet avoidance, low engagement with hygiene education, and menstruation-related discomfort, reveal socio-cultural and resource-related barriers that hinder the application of hygiene knowledge, particularly where privacy, reliability, and supplies are limited. Demographic and contextual differences by gender, age, grade, and school quintile highlight priority groups and locations for support, particularly lower-quintile schools. The modelling further shows that knowledge and attitudes influence hygiene practices, although they are insufficient without enabling school environments, operational systems, and consistent access to hygiene resources.

Based on these findings, the following actions are recommended:

- Integrate hygiene education into the curriculum through structured, age-appropriate content and reinforcement activities.
- Guarantee minimum WASH service standards, including reliable water access, soap availability, and privacy-appropriate sanitation facilities.
- Implement routine maintenance and monitoring systems to ensure functionality and accountability.
- Strengthen school-level WASH governance, including dedicated hygiene coordinators and reporting mechanisms.
- Adopt gender-responsive and stigma-aware hygiene strategies, particularly for menstrual hygiene management.
- Prioritise resource allocation to lower-quintile schools and lagging districts to advance equity and align with SDG 6 targets.

These targeted actions can strengthen both behavioural and systemic determinants of hygiene outcomes, contributing to safer learning environments and improved public health.

## Figures and Tables

**Table 1:** Socio-demographic characteristics of high-school learners participating in the WASH KAP study (*n* = 1200).

Characteristic	Category	N (%)
Gender	Male	562 (46.8)
	Female	638 (53.2)
Grade Level	Grade 8	256 (21.3)
	Grade 9	270 (22.5)
	Grade 10	224 (18.7)
	Grade 11	235 (19.6)
	Grade 12	215 (17.9)
Age Group (Years)	12–14	198 (16.5)
	15–16	452 (37.7)
	17–18	430 (35.8)
	19 and above	120 (10)
School Quintile	Quintile 1–2	327 (27.3)
	Quintile 3	293 (24.4)
	Quintile 4	280 (23.3)
	Quintile 5	300 (25.0)
District	Pinetown	570 (47.5)
	Umlazi	630 (52.5)

**Table 2:** Learners’ knowledge of WASH indicators.

Characteristics/Variables	Wrong Answer (Score 0) N (%)	Correct Answer (Score 1) N (%)
Importance of boiling water	671 (55.9)	529 (44.1)
Duration to boil water	697 (58.1)	503 (41.9)
Proper disposal of faeces	640 (53,3)	560 (46.7)
Material for bathing	371 (30.9)	829 (69.1)
Material for anal cleansing	524 (43.7)	676 (56.3)
Importance of cleanliness	755 (62.9)	445 (37.1)
Critical handwashing times	560 (46.7)	640 (53.3)
Importance of handwashing with soap	891(74.3)	309 (25.8)
Self-protection against Gastrointestinal issues	658 (54.8)	542 (45.2)
Protect others against gastrointestinal issues	530 (44.2)	670 (55.8)

Correct responses were scored 1; incorrect 0.

**Table 3:** Learners’ attitudes toward WASH.

Characteristics/Variables	Wrong Answer (Score 0) N (%)	Correct Answer (Score 1) N (%)
Access to clean WASH facilities is critical	188 (15.7)	1012 (84.3)
Water should be safe to drink	111 (9.3)	1089 (90.8)
School provides enough drinking water	529 (44.1)	671 (55.9)
Importance of cleaning after toilet use	113 (9.4)	1087 (90.6)
School’s toilet provides enough privacy	843 (70.3)	357 (29.8)
Toilets are always in good functioning order	916 (76.3)	284 (23.7)
Handwashing facilities should be available	204 (17.0)	996 (83.0)
Sufficient clean water and soap for hand washing	906 (75.5)	294 (24.5)
Frequent hand washing at school	550 (45.8)	650 (54.2)

Higher scores reflect more positive attitudes.

**Table 4:** Learners’ WASH-related hygiene practices at school.

Characteristics/Variables	Wrong Answer (Score 0) N (%)	Correct Answer (Score 1) N (%)
How do you usually drink water at school	545 (45.4)	655 (54.6)
How much water consumption do you take per day while at school?	600 (50.0)	600 (50.0)
How often do you utilise toilet while you are at school?	693 (57.8)	507 (42.3)
Do you attend hygiene education that are offered in your school?	787 (65.6)	413 (34.4)
Do you urinate behind the school toilets?	252 (21.0)	948 (79.0)
Do you clean up after yourself at the school toilets after using them?	411 (34.3)	789 (65.8)
Do you wash your hands after going to the bathroom?	301 (25.1)	899 (74.9)
Do you and your girls’ friends feel comfortable using your school toilets during your menstrual cycles?	919 (76.6)	281 (23.4)
Do you miss school during menstruation period?	615 (51.2)	585 (48.8)
Do you maintain personal hygiene during menstruation while you are at school?	672 (56.0)	528 (44.0)

Higher scores reflect better hygiene behaviour.

**Table 5:** Correlations among WASH knowledge, attitudes, and practices.

	Knowledge	Attitudes	Practice
Knowledge	1	0.15 **	0.27 **
Attitudes	0.15 **	1	0.30 **
Practice	0.27 **	0.30 **	1

** Correlation significant at *p* < 0.01 (2-tailed).

**Table 6:** Associations between learners’ demographic characteristics and WASH knowledge, attitudes, and practices.

Demographics	Knowledge		Attitudes		Practices	
	M	SD	M	SD	M	SD
Gender						
Female	0.50	0.22	0.60	0.19	0.5914	0.19
Male	0.44	0.21	0.60	0.20	0.40	0.16
*t* value	4.83		0.81		18.03	
Sig.	0.001		0.419		0.001	
Age						
12–15	0.47	0.21	0.61	0.20	0.53	0.21
16–19	0.49	0.22	0.59	0.18	0.50	0.20
20–24	0.45	0.23	0.51	0.16	0.5000	0.22
F ratio	2.05		5.60		1.96	
Sig.	0.13		0.004		0.14	
Grade						
8	0.45	0.21	0.60	0.20	0.51	0.21
9	0.46	0.21	0.62	0.21	0.54	0.21
10	0.50	0.22	0.59	0.17	0.53	0.20
11	0.51	0.22	0.62	0.17	0.52	0.20
12	0.48	0.21	0.54	0.18	0.48	0.21
F ratio	3.88		6.21		2.27	
Sig.	0.004		0.001		0.06	
Quintile						
1	0.44	0.16	0.55	0.19	0.50	0.20
2	0.36	0.19	0.51	0.16	0.43	0.20
3	0.49	0.20	0.61	0.18	0.52	0.21
4	0.48	0.21	0.61	0.18	0.50	0.20
5	0.54	0.22	0.63	0.20	0.58	0.20
F ratio	25.02		15.04		18.18	
Sig.	0.001		0.001			0.001
District						
Pinetown	0.44	0.22	0.60	0.20	0.49	0.22
Umlazi	0.51	0.21	0.60	0.18	0.54	0.20
t value	−5.16		0.85		−3.90	
Sig.	0.001		0.40		0.001	

M = mean, SD = standard deviations.

**Table 7:** Bonferroni post hoc multiple comparisons for hygiene practice scores across grade levels.

Grade Comparison	Mean Difference	Standard Error.	Sig. (*p*)	Significant
Grade 8 vs. Grade 9	0.03	0.01	0.01	Yes
Grade 8 vs. Grade 10	0.02	0.01	0.12	No
Grade 8 vs. Grade 11	0.01	0.01	0.46	No
Grade 8 vs. Grade 12	0.02	0.01	0.06	No
Grade 9 vs. Grade 10	−0.01	0.01	0.36	No
Grade 9 vs. Grade 11	−0.02	0.01	0.08	No
Grade 9 vs. Grade 12	−0.03	0.01	0.003	Yes
Grade 10 vs. Grade 11	−0.01	0.01	0.38	No
Grade 10 vs. Grade 12	−0.02	0.01	0.05	No
Grade 11 vs. Grade 12	−0.01	0.01	0.04	Yes

Mean Difference values reflect pairwise post hoc comparisons of grade-level hygiene practice scores. Statistical significance was assessed at *p* < 0.05; “Yes” indicates statistically significant group differences, while “No” indicates non-significant group differences.

**Table 8:** Multiple linear regression predicting WASH-related hygiene practices among learners.

IV	R	R^2^	F	df1; df2	*p*-Value	B (Regression Coefficient)	T	*p*-Value	DV
Knowledge	0.38	0.14	100.25	2; 1197	<0.001	0.23	8.57	<0.001	Practices of good hygiene
Attitudes						0.27	9.83	<0.001	

B = unstandardised coefficient; R^2^ = variance explained; *p* < 0.05 considered significant. Predictors: Knowledge and Attitudes. Outcome: Hygiene Practices. Regression assumptions were satisfied.

**Table 9:** Perceived adequacy of clean water and soap for handwashing across school quintiles (N = 1200).

Does Your School Have Enough Clean Water and Soap for Hand Washing?	Quintile 1	Quintile 2	Quintile 3	Quintile 4	Quintile 5	Total
Negative attitude N (%)	19 (1.6)	164 (13.7)	308 (25.7)	207 (17.3)	208 (17.3)	906 (75.5)
Positive attitude N (%)	11 (0.9)	46 (3.8)	82 (6.8)	63 (5.3)	92 (7.7)	294 (24.5)
Total N (%)	30 (2.5)	210 (17.5)	390 (32.5)	270 (22.5)	300 (25.0)	1200 (100)
X^2^ = 12.08, df = 4, *p* = 0.02						

Values presented as N (%). A chi-square test showed a significant association between school quintile and perceived adequacy of handwashing resources (χ^2^ = 12.08, df = 4, *p* = 0.02).

## Data Availability

The data presented in this study are available on request from the authors.

## Abbreviations

The following abbreviations are used in this manuscript:

**Abbreviation**	**Full Term**
ANOVA	Analysis of Variance
CI	Confidence Interval
df	Degrees of Freedom
DV	Dependent Variable
EHP	Environmental Health Practitioner
HREC	Health Research Ethics Committee
JMP	Joint Monitoring Programme (WHO/UNICEF)
KAP	Knowledge, Attitudes, and Practices
LMICs	Low- and Middle-Income Countries
M	Mean
N	Sample Size
*p*	Probability Value (Significance Level)
SD	Standard Deviation
SDG	Sustainable Development Goal
SPSS	Statistical Package for the Social Sciences
UNICEF	United Nations International Children’s Emergency Fund
UNISA	University of South Africa
WASH	Water, Sanitation, and Hygiene
WHO	World Health Organization
